# Effectiveness of Electrical Stimulation on Upper Limb Function in Children and Young People with Hemiplegic Cerebral Palsy: A Systematic Review

**DOI:** 10.3390/jcm14196718

**Published:** 2025-09-23

**Authors:** Omar Nahhas, Sarah L. Astill, Samit Chakrabarty, Joanna Burdon, Antonio Capozio

**Affiliations:** 1 School of Biomedical Sciences, Faculty of Biological Sciences, University of Leeds, Leeds LS2 9JT, UK; s.l.astill@leeds.ac.uk (S.L.A.); s.chakrabarty@leeds.ac.uk (S.C.); or capozioa@edgehill.ac.uk (A.C.); 2 Health Rehabilitation Sciences, Faculty of Applied Medical Sciences, University of Tabuk, Tabuk 71491, Saudi Arabia; 3 Leeds Teaching Hospitals NHS Trust, Leeds LS9 7TF, UK; joanna.burdon@nhs.net; 4 Department of Psychology, Edge Hill University, Ormskirk L39 4QP, UK

**Keywords:** cerebral palsy, hemiplegic cerebral palsy, electrical stimulation, upper limb function, motor function

## Abstract

**Objectives**: This review seeks to evaluate the effectiveness of electrical stimulation (ES) in improving upper limb function in children and young people (CYP) with hemiplegic cerebral palsy (HCP). **Methods**: A systematic literature search from inception until May 2025 was conducted. Various study designs comparing the effect of different ES techniques such as functional electrical stimulation (FES), transcutaneous electrical nerve stimulation (TENS), neuromuscular electrical stimulation (NMES), transcutaneous spinal cord stimulation (TSCS), and transcranial direct current stimulation (tDCS) on upper limb function in CYP with HCP were included. **Results**: Eighteen studies were selected for review and quality assessment, comprising twelve randomised controlled trials (RCTs) and six non-RCTs. FES was shown to improve upper limb function, though more rigorous and controlled research is needed. Both TENS and NMES demonstrate potential to improve upper limb function, particularly when combined with other interventions. The analysis suggests that variability in reporting tDCS outcomes hinders assessment of its potential benefits for improving upper limb function. **Conclusions**: Current research suggests ES may support upper limb rehabilitation in CYP with HCP, though the overall evidence remains limited. Most studies are small, underpowered, and lack long-term follow-up, limiting confident conclusions. ES should therefore be applied cautiously and only as part of a comprehensive rehabilitation plan.

## 1. Introduction

Cerebral palsy (CP) is a group of sensorimotor disorders that affect the developing foetus or infant brain, influencing motor control ability [1]. CP is considered the most common motor disability in children, with the estimated prevalence worldwide being 2.11 per 1000 live births [2]. Most CP cases present with spastic motor impairment [3]. Spastic hemiplegic CP (HCP) primarily impacts one side of the body. It typically affects the upper limb more than the lower limb, and the distal muscles more than the proximal ones [4,5]. Arm and hand functions, such as reaching, holding, and manipulating objects, are commonly affected [1,6]. Arner et al. [6] found that 60% of children diagnosed with HCP exhibit limited hand function.

There is currently no cure for CP. Upper limb function can be improved through multiple rehabilitation approaches, such as physical and occupational therapy. These may involve strengthening exercises, functional exercises, facilitation and inhibition of muscle tone, and other therapy approaches, such as non-invasive electrical stimulation (ES), and are often employed to augment rehabilitation [7,8,9,10,11]. Non-invasive ES techniques include transcranial direct current stimulation (tDCS), transcutaneous electrical nerve stimulation (TENS), functional electrical stimulation (FES), neuromuscular electrical stimulation (NMES), and transcutaneous spinal cord stimulation (TSCS). Non-invasive ES modalities differ in their target structures, stimulation parameters, application methods, and underlying physiological mechanisms. For instance, FES and NMES primarily target muscle fibre recruitment [12,13,14]; TENS acts on sensory nerves at the peripheral and spinal levels [15]; and techniques like tDCS and TSCS aim to modulate spinal or cortical activity, thereby promoting neuroplasticity [16,17,18], improving movement quality, and reducing spasticity [15,19,20]. Although these approaches play an important role in inducing physiological changes, the efficacy of rehabilitation depends on translating those effects into improved overall function. Therefore, all non-invasive ES modalities share a common therapeutic purpose: to promote functional improvement, enhance motor performance, and eventually improve independence and participation in daily life activities.

NMES [12] and FES [13,14] are non-invasive applications of ES aimed at inducing skeletal muscle contractions. While both techniques stimulate the muscles, NMES is used for muscle rehabilitation, including the prevention of muscle atrophy and the enhancement of muscle strength. In contrast, FES is applied to enhance muscle activation for the restoration of motor functional activities, like grasping [13,14]. Although the term FES became widely used in the rehabilitation literature during the late 20th century, its application remains relevant in those with CP [21]. In FES, electrical impulses are applied directly to peripheral muscles or nerves to activate muscle contractions that support functional activities and improve movement patterns [21]. FES continues to be the focus of active research, with recent reviews supporting its value and efficacy as a promising lower limb rehab intervention, despite acknowledged methodological limitations in existing studies [22]. TENS is a non-invasive application of ES that targets sensory nerves for multiple purposes, such as decreasing spasticity, possibly through modulating motoneuron excitability and the reflex threshold via modified synaptic input which leads to afferent synaptic changes [15,19,20]. In contrast, tDCS is a non-invasive technique in which a weak direct current is applied to the scalp [16]. It is suggested that tDCS influences motor cortex activity, through the inhibition or facilitation of cortical excitability, thereby promoting neural plasticity, which in turn can promote changes in motor control in children and young people (CYP) with CP [23,24]. Finally, TSCS is a non-invasive method of transcutaneous stimulation of the spinal cord, which can be applied as a neuromodulation approach [17,18]. While recent work shows that TSCS improves lower extremity function, the mechanisms underlying functional recovery following TSCS in HCP remain uncertain [25].

Several systematic reviews [26,27,28] have been published recently focusing on the use of ES for improving motor function, such as standing, running, and jumping functions, balance, gait spasticity, or upper limb function in young adults and children living with HCP. However, none of these reviews specifically focused on upper limb and hand function in relation to specific topographical subtypes of HCP. The inclusion in previous reviews of varied subtypes such as diplegia, quadriplegia, ataxia, or cases where the subtype was not reported likely affects the conclusions, as each presents different prognostic outcomes. The focus of this review is on HCP participants who have only received ES and/or have physical and occupational therapy so as to avoid impacting the conclusion by using other interventions, such as dynamic bracing, robot-assisted therapy, or virtual reality. These other interventions have been reported to play a role in improving the affected upper limb’s function, even without ES [29,30]. Finally, to date, none of the published reviews have been comprehensive enough to include a range of non-invasive ES that targets different levels of the motor system such as the brain, spinal cord, and the peripheral part for the upper limbs and hands. It remains crucial to determine the specific contributions of non-invasive ES in combination with physical and/or occupational therapy to improve upper limb function after HCP. This is particularly important as it is frequently recommended in the literature and commonly included in research protocols and widely used in clinical practice, despite the lack of consistent guidelines or conclusive evidence of its efficacy.

### Research Aim and Research Questions

The aim of this systematic review is to critically evaluate and compare the effectiveness of different non-invasive ES modalities (tDCS, TENS, NMES, FES, TSCS), delivered alone or in combination with physical and/or occupational therapy, in improving upper limb function in CYP with HCP; in addition, the review will clarify the current evidence, identify promising approaches for future research, and highlight gaps to guide future clinical application and research. Research Question: Which non-invasive ES modality (tDCS, TENS, NMES, FES, TSCS), delivered alone or as an adjunct to physical/occupational therapy, yields improvements in upper limb function in CYP with HCP?

This review is structured as follows. Section 2 (Methods) describes the search strategy, eligibility criteria, study selection process, synthesis approach, and appraisal of methodological quality. Section 3 (Results) presents the main findings, including study characteristics, types of electrical stimulation interventions, outcome measures, and methodological quality assessment. Section 4 (Discussion) interprets the results in relation to the existing evidence, addresses practical implications, outlines limitations, and highlights directions for future research. Finally, Section 6 (Conclusion) summarises the key insights of the review.

## 2. Materials and Methods

### 2.1. Search Strategy

A comprehensive search was conducted from inception to May 2025. The following databases were used to conduct literature searches: CINAHL, Cochrane, EMBASE, PEDro, PsycINFO, PubMed, Scopus, Ovid Medline, and Web of Sciences. To structure the review question and identify search terms and their synonyms, the population, intervention, comparison, outcomes, and study (PICOS) framework was applied. Furthermore, a number of search terms were used to identify relevant studies, and the Boolean operators “AND” and “OR” were used to combine keywords. This review was registered with the International Prospective Register of Systematic Reviews (PROSPERO; registration number CRD42023414421). The full description of the search strategy used and the abstract checklist are provided in the Supplementary Materials.

### 2.2. Eligibility Criteria

#### 2.2.1. Inclusion Criteria

- Participants aged 2 to 21 years old, both males and females. The lower limit reflects the age when CP-related motor impairments are most consistently recognised in clinical practice and diagnosed [31,32,33]. The upper limit extends into adolescence and early adulthood, when neuroplasticity and motor skill development are still responsive to rehabilitation. Additionally, the upper limit is consistent with current evidence and clinical practice in both research and clinical settings [34,35,36].
- Diagnosis of HCP.
- English studies published of any design (except case studies with fewer than 3 participants).
- Studies utilising one of the following ES modalities: FES, TENS, NMES, TSCS, or tDCS.
- Studies utilising ES plus physical therapy, occupational therapy, or ES alone.
- Physical and occupational therapy including exercises (strength, range of motion, stretching), functional exercises (sensorimotor training, task-oriented training), and facilitation and inhibition of muscle tone (neurodevelopmental treatment) [7,8,9,10,11].
- Upper limb and hand function outcomes (including fine and gross motor skills, range of motion, muscle strength, functional grip and release, isolated finger movements, protective responses, weight-bearing capacity, object manipulation, movement fluidity, placement accuracy, and performance of daily activities).

#### 2.2.2. Exclusion Criteria

- Studies including mixed CP or other neurological disorder subtypes without subgroup analysis for the HCP population.
- Participants younger than 2 years or older than 21 years.
- No confirmed diagnosis of HCP.
- Non-English publications.
- Grey literature (e.g., dissertations, conference abstracts, preprint reports not peer-reviewed).
- Case studies with fewer than three participants.
- Studies not utilising any of the following ES modalities: FES, TENS, NMES, TSCS, or tDCS.
- Studies using ES combined with interventions other than physical therapy, occupational therapy, or ES alone.
- Studies without upper limb or hand function outcomes.

### 2.3. Study Selection

Two reviewers, O.N. and A.C., independently screened the titles and abstracts of studies based on the eligibility criteria to identify relevant research. They also extracted critical information from these studies, including author names, study designs, participant demographics, intervention protocols (including parameters, session and trial durations, and follow-up strategies), countries of origin, outcomes, and a summary of the results. The study selection process was managed using EndNote reference management software 20, which was used to organise citations, remove duplicates, and facilitate collaboration between reviewers.

### 2.4. Study Synthesis

Narrative synthesis was conducted as an alternative to meta-analysis owing to considerable heterogeneity across designs and interventions, limited and imbalanced sample sizes, and missing data required for effect-size estimation.

### 2.5. Study Appraisal

The quality of the included studies was assessed independently by two reviewers (O.N. and A.C.) using a modified version of the Cochrane risk of bias (RoB) tool 2 (see Supplementary File S1). Where there were differences in the ratings given by the two reviewers, a discussion took place with a third party (S.A.), and a consensus rating was agreed. The risk-of-bias tool assesses risk based on five domains: (1) bias arising from the randomisation process assessed through study design, random sequence generation, allocation concealment, and baseline differences; (2) deviations from intended interventions assessed through the blinding of participants, blinding of personnel, deviations from intended intervention, effect-of-assignment analyses, and concurrent interventions or unintended exposures; (3) missing outcome data assessed through completeness and handling of outcome data; (4) bias in the measurement of the outcome, assessed through appropriateness of outcome measurement and blinding of outcome assessment; (5) bias in the selection of the reported result assessed through the selective reporting of outcomes. Each domain was rated according to its potential risk of bias, which was indicated as either high, low, some concern, or non-applicable. Risk of bias was visualised using the standard Robvis (risk-of-bias visualisation) template: This generates a traffic-light plot across the following domains: random sequence generation, allocation concealment, blinding of participants and personnel, blinding of outcome assessment, incomplete outcome data, selective reporting, and other sources of bias [37,38].

## 3. Results

### 3.1. Search Results

**Figure 1:**
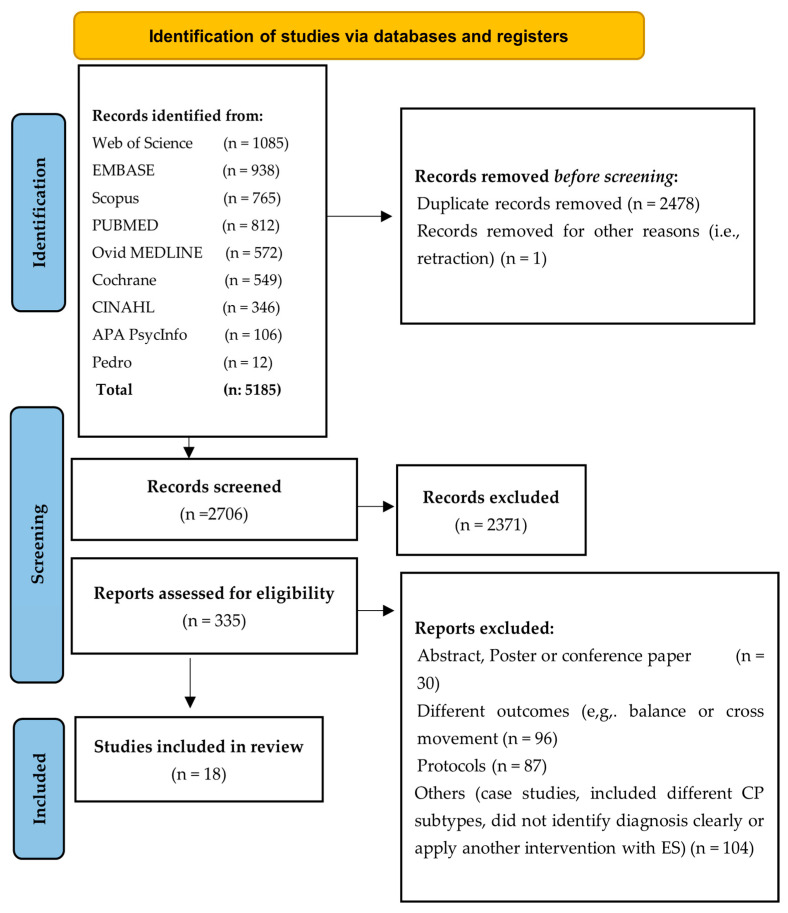
PRISMA flow diagram.

The initial search yielded 5185 studies. To eliminate duplicate references, a manual review was conducted to identify and exclude duplicates that were not detected by the citation manager. A total of 2479 studies were excluded using both the citation manager and manual review processes. Next, the screening process was carried out on 2706 studies based on their title and abstract, and 2371 studies were excluded. Following this screening phase, 335 studies were eligible for full screening. As a result of applying the eligibility criteria, 18 studies were selected for review and quality assessment. Figure 1 presents a flow diagram of the PRISMA search process for electronic databases. 

### 3.2. Study Characteristics

A variety of study designs have been included in this review. Eleven studies [39,40,41,42,43,44,45,46,47,48,49] were RCTs and seven non-RCTs [50,51,52,53,54,55,56]. The selected studies were conducted in various geographic areas, including China [42,45], the United States [41,50,56], the United Kingdom [55], India [40,44], Serbia [51], Saudi Arabia [39], Brazil [43], Finland [53], Iran [46,52], Spain [48], Canada [47], and Egypt [49]. The total number of participants included in the systematic review was 538, with a range between 8 [51] and 83 [47] participants per study. The participants’ ages were between 2 [45] and 21 [41] years old. The characteristics of selected studies, including study design, country, participant demographics, and distribution of manual ability levels, are provided in Table 1.

### 3.3. Electrical Stimulation

#### 3.3.1. tDCS Interventions

Nine studies employed tDCS [41,42,43,46,47,48,50,52,56], and all studies applied tDCS stimulation for 20 min per session. While seven of these studies [41,43,46,47,48,52,56] combined tDCS with other interventions such as training for functional activities and motor skill development, two studies [42,50] applied tDCS alone. The frequency of sessions varied, with three studies [41,47,56] conducting daily sessions for ten days, and the other studies [42,43] conducting a single session each. One study [46] included 20 sessions, another [48] had 13 sessions, and one study [50] involved five sessions (three active tDCS sessions, one sham, and one mock). Another study [52] implemented four sessions, each with a different condition: a-tDCS-offline, a-tDCS-online, s-tDCS-offline, and s-tDCS-online. The stimulation intensity also varied, ranging from 1.5 mA in four studies [42,46,50,56] to 0.7 mA [41] and 1 mA in the remaining studies [43,47,48,52].

#### 3.3.2. FES Interventions

Three studies [51,54,55] applied FES alongside neurodevelopmental treatment in one study [51] and alongside strengthening exercises in another [54]. Meanwhile, a third study [55] applied FES alone. The frequency of FES ranged from 30 Hz [54,55] to 50 Hz [51], with a pulse width of 300μs across all studies [51,54,55]. These studies scheduled 24 sessions (3×/week over 8 weeks) [54], 15 sessions (5×/week over 3 weeks) [51], or daily sessions for 6 weeks [55]. In these studies, intensity levels were adjusted to between 10 mA and 40 mA [51,55].

#### 3.3.3. TENS Interventions

Two studies [40,44] applied TENS in conjunction with interventions such as sensorimotor and task-oriented training, while another study [39] combined TENS with exercises, including grip strength and stretching. TENS was used in three studies [39,40,44], with stimulation durations ranging from 30 [39] to 60 [40,44] minutes. These studies [39,40,44] scheduled 24 (3×/week over 8 weeks) sessions each, employing a frequency of 100 Hz and different intensities based on patient feedback [40,44] or specific parameters (i.e., 50 mA) [39].

#### 3.3.4. NMES Interventions

Three studies [45,49,53] applied NMES in conjunction with other PT interventions such as neurodevelopmental treatment [49,53] and activities of daily living [45], with session lengths from 20 min [45,49] to 20–40 min [53] and frequencies of 30 [49] to 50 Hz [45]. The intensities employed ranged between 2 and 100 mA or until tolerated. These studies [45,49,53] scheduled 10 [45], 12 [53], and 36 [49] sessions.

#### 3.3.5. TSCS Interventions

No studies were identified that applied TSCS as an intervention for CYP with HCP. While interest in tSCS for CP more broadly is emerging, and early feasibility work is beginning to appear in CYP with other CP subtypes, we found no eligible studies focused specifically on HCP. As a result, there is currently no direct evidence to inform the effectiveness or optimal stimulation parameters of tSCS for CYP with HCP. Table 2 provides a summary of the stimulation parameters applied across the included studies, showing variations in intensity, frequency, and duration.

### 3.4. Outcome Measures Summary

Across the research studies included in our analysis on the effectiveness of non-invasive ES, researchers utilised many different assessment tools. Among these, the most frequently used were: the box-and-blocks test (BBT) [42,46,47,50,56], the Quality of Upper Extremity Skills Test (QUEST) [40,44,51], the Jebsen–Taylor hand function test (JTHFT) [39,47,54,55], a dynamometer [39,41,52,54] for measuring muscle strength, the ABILHAND-Kids questionnaire [39,44,56] for assessing manual ability in children, the assisting hand assessment (AHA) [41,47,56], a goniometer [45,51,52], the nine-hole peg test (9HPT) [44,45], and the Peabody developmental motor scales (PDMS) [45,49]. Less commonly used tools, employed in one study each, included the Melbourne-2 [42], Selective Control of the Upper Extremity Scale (SCUES) [42], upper arm-movement kinematics [43], Zancolli classification [53], a modification of the PC system [55], Daniels and Worthingham’s muscle test [53], an Upper Extremity Function Test (UEFT) [45], sphygmomanometry [45], Fugl-Meyer assessment [46], Bruininks–Oseretsky test [46], and Shriners Hospital Upper Extremity Evaluation (SHUEE) [48].

#### 3.4.1. Upper Extremity Function and Hand Skills

Of the three studies [40,44,51] employing QUEST to assess upper extremity skills, two studies [40,44] reported significant improvements in the group receiving TENS combined with sensorimotor task-oriented training, compared to their control groups. Additionally, FES [51] significantly improved arm and hand function, although there was no control group to compare to. Studies that used JTHFT [39,47,54,55] provided varied outcomes that ranged from no significant improvement after applying FES [54], TENS [39], and tDCS [47] to significant enhancements in hand function following FES [55]. The AHA test was used in three studies [41,47,56] and the ABILHAND-Kids questionnaire was used in three studies [39,44,56]. The findings from all these studies [39,41,44,47,56] indicated no significant effect on upper extremity and hand skills for the tDCS [41,47,56] group and TENS [39,44] group. In addition, tDCS [42] did not lead to significant improvements in upper extremity function when assessed with SCUES [42], the Melbourne-2 test [42], or SHUEE [48]. In contrast, a significant enhancement in hand function was observed with the Zancolli classification after NMES [53]. Both the Fugl-Meyer assessment and the Bruininks–Oseretsky test indicated significant improvement following tDCS and occupational therapy (OT) [46].

An Upper Extremity Functional Test (UEFT) demonstrated that participants who received constraint-induced movement therapy (CIMT) combined with NMES showed significant improvements in arm and hand function [45]. Satheeskumar et al. [44] reported that TENS combined with sensorimotor task-oriented training significantly enhanced fine motor skills, as measured by improvements in the nine-hole peg test (9HPT). In contrast, Xu et al. [45] reported that NMES combined with constraint-induced movement therapy did not enhance fine motor skills, as measured by 9HPT. BBT was used to assess unilateral manual dexterity in five tDCS studies [42,46,47,50,56]. These studies [42,46,47,50,56] reported varied outcomes, ranging from no significant improvement in three studies [47,50,56] to significant enhancements in two studies [42,46]. Finally, PDMS was used to assess grasping abilities following NMES [45,49] application in two studies. Xu et al. [45] reported significant improvements in grasping abilities after NMES application and at follow-up, compared to the outcomes from two control groups. In contrast, Sarhan et al. [49] reported no significant improvement in PDMS in the experiential group compared with the control group.

#### 3.4.2. Muscle Strength

The effectiveness of ES interventions in enhancing muscle strength was assessed using the Daniels and Worthingham test [53], dynamometers [39,41,52,54], and sphygmomanometry [45]. Mäenpää et al. [53] demonstrated that applying NMES led to significant improvements in arm flexion and extension strength with the Daniels and Worthingham test, particularly in younger children (i.e., less than 4 years old). Xu et al. [45] used sphygmomanometry to assess muscle strength changes after applying NMES combined with CIMT, showing significant post-intervention improvement which was maintained at follow-up compared to the control group. Two studies [39,54] demonstrated significant hand grip strength improvements using TENS [39] and FES [54], measured with handheld and Microfet-2 dynamometers, respectively. In contrast, one tDCS study [41] showed no significant grip strength changes, as assessed by a handheld dynamometer, while Farzamfar, Heirani [52] reported significant increases in hand grip strength under tDCS-offline, sham tDCS-offline, and sham tDCS-online conditions. In contrast, a decline in strength was reported in the tDCS-online condition.

#### 3.4.3. Other Rehabilitation Outcomes (Kinematics and Range of Motion)

**Figure 2:**
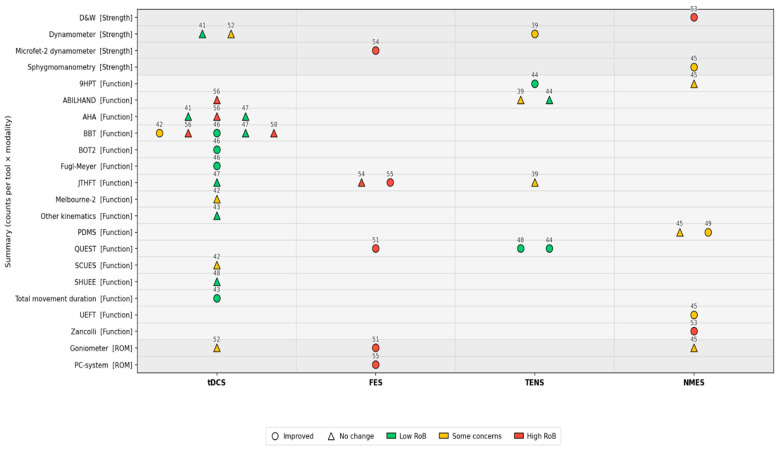
Summary of improvements across outcome tools and ES modalities. Each marker shows a study cohort for a given tool × modality (tDCS, FES, TENS, NMES); circles = improvement, triangles = no change. Marker colour = risk of bias (green = low, yellow = some concerns, red = high). Outcomes are grouped by domain.

**Figure 3:**
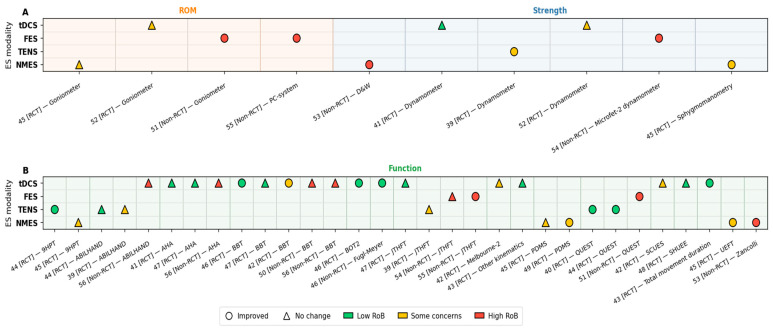
Study results by domain and ES modality. Panel (**A**): Strength + ROM. Panel (**B**): Function. Each point represents a study outcome pair for a given modality. Circles represent improvement; triangles represent no change. Marker colour indicates risk of bias (green = low, yellow = some concerns, red = high).

Other tools and outcomes include the use of upper arm movement kinematics by Moura et al. [43], who found significant reductions in total time to complete the reaching task in both paretic and non-paretic limbs for the tDCS group. In addition, significant improvements in the range of motion (ROM) of the wrist were noted across four studies [45,51,52,55]. ROM was assessed with a goniometer in one study investigating the use of tDCS [52], FES [51], and NMES [45] and a PC system (ROM assessment) for a further study using FES [55]. The ROM increased significantly in every group, including those with and without ES; however, no significant between-group differences were detected [45]. However, one study [52] reported that significant improvements in ROM were observed under the tDCS-offline, sham tDCS-offline, and sham tDCS-online conditions, while no improvement or a decrease was noted in the tDCS-online condition. Finally, two studies [51,55] reported increasing ROM. The main characteristics of the studies, ES type, outcome measures, and summary are provided in Table 3. Figure 2 and Figure 3 show that outcomes grouped into domains such as strength, motor control, and function most frequently demonstrated improvements with ES. Figure 2 summarises the evidence across tools and modalities, indicating where improvements were most consistently reported. Figure 3 presents individual study outcomes together with risk-of-bias indicators.

### 3.5. Methodological Quality

**Figure 4:**
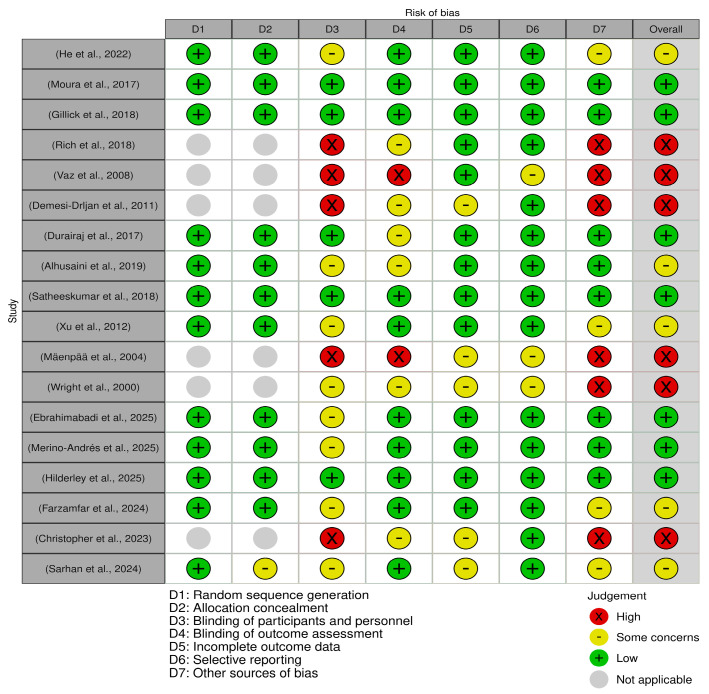
Risk of bias summary for included studies. Judgments across seven domains of bias are presented for each study: random sequence generation (D1), allocation concealment (D2), blinding of participants and personnel (D3), blinding of outcome assessment (D4), incomplete outcome data (D5), selective reporting (D6), and other sources of bias (D7). The symbols indicate the level of risk: green (+) = low risk, yellow (–) = some concerns, red (×) = high risk, and grey ( ) = not applicable. Studies: (Alhusaini et al., 2019) [39]; (Durairaj et al., 2017) [40]; (Gillick et al., 2018) [41]; (He et al., 2022) [42]; (Moura et al., 2017) [43]; (Satheeskumar et al., 2018) [44]; (Xu et al., 2012) [45]; (Ebrahimabadi et al., 2025) [46]; (Merino-Andrés et al., 2025) [48]; (Hilderley et al., 2025) [47]; (Sarhan et al., 2024) [49]; (Christopher et al., 2023) [50]; (Demesi-Drljan et al., 2011) [51]; (Farzamfar et al., 2024) [52]; (Mäenpää et al., 2004) [53]; (Vaz et al., 2008) [54]; (Wright et al., 2000) [55]; (Rich et al., 2018) [56].

Table 4 provides a comprehensive overview of the methodological quality of the included studies. Of the 18 studies, the overall quality was rated as 7 low risk [40,41,43,44,46,47,48], 6 high risk [50,51,53,54,55,56], and 5 with some concerns [39,42,45,49,52]. The most frequent limitations were related to inadequate blinding of personnel, participants, and outcome assessors, as well as weaknesses in study design. In contrast, several domains were consistently strong: outcome measurement was rated low risk in all studies, selective reporting was mostly low risk, and only minimal issues were observed with deviations from the intended intervention and concurrent interventions. Random sequence generation was also generally low risk where applicable. Figure 4 presents a traffic light risk-of-bias summary for the included studies.

## 4. Discussion

This systematic review analysed 18 studies of CYP with HCP (N = 538) and presented information on the effects of different types of ES including FES, TENS, NMES, and tDCS on upper limb function in CYP with HCP. In addition, no studies were identified that applied TSCS as an intervention. Of the 18 studies, the overall quality was rated as 7 low risk [40,41,43,44,46,47,48], 6 high risk [50,51,53,54,55,56], and 5 with some concerns [39,42,45,49,52]. The review aimed to address the research question: Which non-invasive ES modality (tDCS, TENS, NMES/FES, or TSCS), delivered alone or as an adjunct to physical/occupational therapy, yields improvements in upper limb function in CYP with HCP?

### 4.1. Transcranial Direct Current Stimulation

The effects of tDCS on upper limb function in CYP with HCP show variable outcomes across different studies [41,42,43,46,47,48,50,52,56]. Three studies [42,43,52] focusing on a single session of tDCS reported improvements in hand movement reaction times, as measured by the BBT [42], and in the time to complete a reaching task, according to kinematic assessments [43]. However, these positive findings were not supported by other measures such as the SCUE [42], Melbourne 2 assessment [42], and kinematic variables [43] like velocity, precision, and smoothness, which showed no significant changes. Another study [52] implemented one session and then tested both offline and online tDCS modes applied under different conditions. Improvements in ROM and grip strength were recorded in both the tDCS and sham-tDCS groups [52]. The quality of the studies varied, as one [43] exhibited a low risk of bias whereas others [42,52] had some concerns. Importantly, none of the three trials [42,43,52] included long-term follow-up: all focused solely on the immediate effects.

On the other hand, five studies [41,47,48,50,56] involving multiple sessions indicated that there was no significant improvement except one study that indicated improvement in all the outcome measurement tools after having 20 sessions [46]. The study quality ranged from low risk of bias [41,46,47,48] to high risk of bias [50,56]. These results are consistent with recent systematic reviews and meta-analyses in stroke patients [57] which showed that multiple tDCS sessions did not significantly impact upper limb function. However, in healthy adults, tDCS significantly improved motor performance metrics such as reaction time and task completion time [58]. These contrasts highlight the need for further research to understand the mechanisms behind the short-term improvements observed with tDCS in CYP with CP, potentially providing optimised or combined therapies for maximum therapeutic effect.

It is important to note that a limitation across the HCP tDCS studies is the heterogeneity in stimulation protocols, including current intensity, session number, duration, and concurrent tasks, which limits comparisons and may contribute to variability in treatment response. In this review, stimulation ranged from 0.7 mA (single session) to 1.5 mA (20 sessions), with only the highest dose study [46] reporting consistent improvements, though without follow-up data to assess long-term effects. Therefore, future research should systematically clarify the dose–response relationship by directly comparing specific parameters, such as stimulation intensities (0.7 vs. 1.5 mA), session numbers (fewer than 20 vs. ≥20 sessions), and clearly defined session durations, with appropriate sham control and follow-up assessments.

### 4.2. Functional Electrical Stimulation

Three studies [51,54,55] applied FES, and all reported significant improvements in all outcome measurements (i.e., dynamometer, QUEST, goniometer and PC system), except for one tool, JTHFT. Although the results were promising, these studies [51,54,55] were limited by methodological issues, as they all had a high risk of bias.

Specifically, JTHFT was used in two different studies [54,55]; it showed significant improvement in one study [55], but not in another [54]. These contradictory results could potentially be due to a difference in the study design: daily FES sessions were employed in [55], while sessions three times per week were employed in [54]. This is consistent with our summary that effective protocols tended to use daily or five-times-weekly schedules over 3–6 weeks. Accordingly, session frequency appears to underlie the JTHFT discrepancy. Given these findings, there is a clear need for more rigorous and controlled research protocols to better determine the role of FES in enhancing hand function.

A review of stimulation sessions indicates that all effective protocols clustered around 30–50 Hz, 300 µs pulses for ≥30 min per session daily or 5 times over 3–6 weeks; lower-frequency protocols were less consistently beneficial. Due to the relatively high risk of bias, however, these findings must be interpreted with caution, and a future robust trial design is urgently needed.

### 4.3. Transcutaneous Electrical Nerve Stimulation

Three studies [39,40,44] have shown that TENS can improve hand and upper limb function. It has been consistently observed across these studies [39,40,44] that there is a significant improvement in most outcome measurement tools such as QUEST, dynamometer, and 9HPT. However, an exception is the ABILHAND-Kids scale, used in two studies [39,44], which did not show significant improvement. The ABILHAND-Kids scale assesses the use of both hands in complex multi-joint tasks that simulate active daily life, such as dressing and opening containers [59]. A potential reason for the lack of significant improvements in the ABILHAND-Kids scale could be that the training exercises in studies [39,44] primarily focused on using one hand, whereas the ABILHAND-Kids scale is predominantly a bimanual measurement tool [59]. Additionally, the methodological quality of these studies varied, with specific concerns about the blinding of the outcome assessor raised in two studies [39,40] and low risk in one study [44]. Despite the mixed quality of the studies, the evidence suggests that TENS has the potential to enhance upper limb function.

All three TENS protocols utilised a consistent, similar approach, employing parameters of 100 Hz frequency, 200–250 µs pulse width, administered for 60 min, three times per week over 8 weeks. This convergence indicates that clinicians may consider these parameters as a valid starting point when implementing TENS alongside hand function training for patients with HCP. However, it is important to note that only three studies have examined TENS use for the upper limb in HCP. Of these, two raised concerns, while one reported a low risk of bias. These findings suggest that although the parameters appear generally reliable, clinicians should proceed with caution and monitor patient response closely when incorporating TENS into the treatment plan.

### 4.4. Neuromuscular Electrical Stimulation

The effects of NMES on upper limb function in CYP with CP show potential to enhance hand and upper limb function. Across three studies [45,49,53] that applied NMES, participants demonstrated significant improvement in most outcome measurements such as sphygmomanometry [45], UEFT [45], PDMS [49], Daniels and Worthingham [53], and Zancolli classification [53], except for the range of motion, which was measured by a goniometer, and manual dexterity, measured by the 9HPT test [45] and PDMS [45]. All studies [45,49,53] combined NMES with additional interventions, such as constraint-induced movement therapy [45], neurodevelopmental techniques [53] and PT [49]. Moreover, the methodological quality of these studies varied, with concerns raised in two studies [45,49] due to the lack of participant blinding, and another [53] was considered high risk due to several issues such as study design and blinding of participants.

According to Yıldızgören et al. [60], applying NMES alongside conventional exercises and hand orthosis improved the active wrist range of motion, measured by a goniometer, and hand function in children with CP. Therefore, applying NMES and using a hand orthosis might be useful for maximising the improvement of the wrist range of motion. A different study [61] applied NMES with hand CP treatments where group 1 had daily NMES sessions, group 2 used daily dynamic bracing, and group 3 combined both treatments, with each lasting 30 min twice a day. The intervention lasted for six months in all groups and was applied only to the affected extremity. Statistically significant differences were found in all three measures, the Melbourne test, dynamometer, and Zancolli classification, but only for those treated with the combined NMES and hand orthosis [61]. Therefore, NMES has been utilised in combination with other interventions, and all these studies [45,49,53,60,61] have indicated improvements in hand function. However, the question still arises regarding the most effective combination of interventions. Future research should prioritise RCTs that directly compare NMES combined with hand orthoses versus NMES alone to determine whether the addition of orthotics provides a significant functional improvement.

### 4.5. Transcutaneous Spinal Cord Stimulation Interventions

No studies to date have applied tSCS specifically as an intervention for CYP with HCP. While there is growing interest in the use of tSCS for CP more broadly, and early feasibility studies have begun to emerge in CYP with other CP subtypes, we found no eligible studies focused exclusively on HCP.

Although still in the early stages of clinical investigation, several pilot studies [17,18] involving approximately 40 children have reported promising results. tSCS has shown preliminary evidence of effectiveness in improving motor function in children with CP. Participants demonstrated significant improvements in functional activities such as transitioning from sitting to standing, maintaining an upright seated posture, and stepping on a treadmill. These improvements were reflected in both clinically and statistically significant gains on the Gross Motor Function Measure-88 (GMFM-88) [62], which assesses five dimensions of gross motor function: lying and rolling, sitting, crawling and kneeling, standing, and walking/running/jumping.

Despite these early findings, the underlying mechanisms through which tSCS benefits children with CP remain poorly understood. Some evidence suggests functional improvements may be linked to enhanced neuromodulation [17,18,63,64]. However, the current evidence base is limited by small sample sizes and a lack of RCTs. More research is needed to establish the long-term safety, efficacy, and optimal parameters of tSCS in CYP with HCP.

Importantly, the effectiveness of tSCS for CYP with HCP, particularly those experiencing difficulties with hand and arm function, has yet to be established. Overall, there is currently no direct evidence to inform the efficacy or optimal stimulation parameters of tSCS specifically for CYP with HCP.

### 4.6. Practical and Future Research Recommendations

- tDCS is not currently recommended for routine clinical use in CYP with HCP. If applied, it should be limited to research or closely monitored settings, paired with motor practice, and include both immediate and follow-up assessments. Future studies should explore dose–response effects (e.g., 0.7–1.5 mA and session number).
- FES may support hand function improvement in CYP with CP, particularly when applied daily using parameters around 30–50 Hz, 300 µs pulse width, for at least 30 min per session over 3–6 weeks. However, all supporting studies had a high risk of bias, and outcomes varied with application frequency. Well-designed, controlled trials are needed to determine optimal dosing and confirm efficacy.
- TENS may be considered as an adjunct to upper limb training in CYP with HCP, using standardised parameters (100 Hz, 200–250 µs, 60 min, three times weekly for 8 weeks), especially for unilateral function. Outcome measures may include QUEST, 9HPT, and dynamometry. Given limited effects on bimanual outcomes (e.g., ABILHAND-Kids), future studies should combine TENS with bimanual tasks with follow-up periods.
- NMES may improve upper limb function in CYP with CP, particularly when combined with interventions such as hand orthoses or constraint-based therapy. Future RCTs should compare NMES alone versus NMES combined with orthoses to evaluate the added benefit.
- TSCS: Feasibility and pilot studies are needed to evaluate the use of transcutaneous spinal cord stimulation (TSCS) for upper limb goals in CYP with HCP, focusing on safety, acceptability, and preliminary efficacy given the current evidence gap.

## 5. Limitations

To the authors’ knowledge, this is the first systematic review to examine the effects of ES delivered alone or in combination with physical and occupational therapy on upper limb function in CYP with HCP. No studies have examined TSCS in CYP with HCP, and while early feasibility work exists in other CP subtypes, there is no direct evidence to guide its use or dosing in this population. This review focused mainly on English-language papers, excluding studies from the grey literature, which could potentially limit its accuracy and applicability. The non-English-language papers were excluded due to a lack of funding for translation and the possibility of language bias. The grey literature was excluded by design to prioritise peer-reviewed evidence and reduce heterogeneity from variably reported, non–peer-reviewed sources. Although the grey and unpublished literature can broaden the evidence base, their exclusion may increase the risk of publication bias, and the direction and magnitude of effects in this review may therefore be inaccurately estimated. It is also less appropriate to build recommendations on work that lacks peer review. However, these sources can be useful in suggesting directions for further research.

It is important to note that the quality of the primary studies included in this review varies from low to high. Due to this, conclusions drawn from this study should be treated with caution until more rigorous RCTs have been conducted. Additionally, it is difficult to apply the results of this review generally to the HCP community because of the limited number of participants (n = 538). Moreover, due to the small number of studies we included, and the variation in whether ES was applied alone or with other rehabilitation modalities, it is difficult to isolate the independent effect of ES from the effects of combined treatment. Additionally, limitations related to participant characteristics should be considered. The broad age range of participants (2–21 years) introduces uncertainty in interpretation, as most studies did not stratify by developmental stage or consider adolescence-specific factors such as developmental milestones, age-related increases in muscle strength, and differences in therapy responsiveness. We recommend that future studies pre-specify age-specific analyses and include adolescence-specific factors where relevant.

## 6. Conclusions

Current studies suggest that ES can support upper limb rehabilitation in CYP with HCP, yet the overall evidence remains uncertain. No studies so far have examined TSCS, suggesting a potential avenue for future research. Single-session tDCS tends to give only temporary improvements in hand reaction time, while longer interventions do not usually lead to noticeable gains in hand function. FES evidence is weak, based on small trials whose designs make their findings hard to interpret with confidence. TENS shows somewhat stronger, though still preliminary, evidence of efficacy. NMES appears effective mainly when paired with hands-on therapy or supportive bracing, suggesting that electricity works best as an added aid rather than a stand-alone treatment. Due to the small size of most trials and the lack of follow-up, and the variation in whether ES was applied alone or with other therapies, the independent effect of ES remains unclear and clear guidance is still lacking regarding the appropriate protocols and the long-term effectiveness of these interventions. To ensure that robustly designed trials answer these questions, future studies should directly compare ES-alone and ES-plus-therapy conditions. For now, ES should be provided with care and considered mainly as part of a comprehensive rehabilitation programme.

## Figures and Tables

**Table 1:** Characteristics of selected studies: design, participants, and setting.

Study	Study Design	Participants’ Details						Country
		Age Range	Number of Participants	Participants’ Distribution		Manual Ability Classification System L: Level		
		Years	N=	Experimental	Control	Experimental	Control	
[42]	RCT	3–6	30	15	15	L: I = 14 L: II = 1	L: I = 14 L: II = 1	China
[41]	RCT	7–21	20	10	10	L: I = 1 L: II = 8 L: III =1	L: I = 1 L: II = 8 L: IV = 1	USA
[43]	RCT	6–12	20	10	10	L: I: = 3 L: II: = 7	L: I: = 1 L: II: = 9	Brazil
[56]	Single-group, multiple-baselines, open-label design	8–19	8	8	-	L: I = 1 L: II = 5 L: III = 2	-	United States
[54]	Pre-test/post-test design	7–11	9	9	-	L: I = 1 L: II = 8	-	Brazil
[51]	Pre-test/post-test design	4–8	13	13	-	L: I = 7 L: II = 3 L: III = 3	-	Serbia
[55]	Pre-test/post-test design	Mean age 10, no range	8	8	-	-	-	United Kingdom
[40]	RCT	4–12	30	15	15	L: II–III		India
[39]	RCT	6–12	29	15	14	L: I–III = 29		Saudi Arabia
[44]	RCT	4–12	60	30	30	L: I = 4 L: II = 18 L: III = 8	L: I = 9 L: II = 14 L: III = 7	India
[45]	RCT	2–14	68	23	45	-	-	China
[53]	Pre-test/post-test design	2–12	12	12	-	-	-	Finland
[46]	RCT	5–10	50	25	25	-		Iran
[48]	RCT	4–8	18	9	9	L: I–III		Spain
[47]	RCT	6–18	83	41	42	L: I–IV		Canada
[49]	RCT	4–5	60	30	30	-		Egypt
[52]	RCT	6–12	10	10	-	L: I–II		Iran
[50]	Pre-test/post-test design	10–19	10	10	-	-		USA

**Table 2:** Summary of stimulation parameters used across included studies.

Study	ES Modality	Freq (Hz)	PW (µs)	Current (mA)	Dose/Exposure Time
[42]	tDCS	-	-	1.5	20 min × 1 session
[41]	tDCS	-	-	0.7	20 min × 10 sessions
[43]	tDCS	-	-	1	20 min × 1 session
[56]	tDCS	-	-	1.5	20 min × 10 sessions
[46]	tDCS	-	-	1.5	20 min × 20 sessions
[48]	tDCS	-	-	1	20 min × 13 sessions
[47]	tDCS	-	-	1	20 min × 10 sessions
[52]	tDCS	-	-	1	20 min × 1 session (each, 1 wk apart × 4 conditions)
[50]	tDCS	-	-	1.5	20 min × 3 sessions
[54]	FES	30	300	until visible contractions	5 s precontraction; 3 sessions/wk × 8 wk (contractions)
[51]	FES	50	300	10–40	15–30 min; 5 sessions/wk × 3 wk
[55]	FES	30	300	10–40	30 min daily × 6 wk
[40]	TENS	100	200	until sensation threshold	60 min; 3 sessions/wk × 8 wk
[39]	TENS	100	250	50	30 min; 3 sessions/wk × 8 wk
[44]	TENS	100	200	until initial current sensation	60 min; 3 sessions/wk × 8 wk
[45]	NMES	50	300	≤100	20 min; 5 sessions/wk × 2 wk
[53]	NMES	40	300	2–10	20–40 min; 12 sessions (over 5 wk)
[49]	NMES	30	300	to tolerate	20 min; 3 sessions/wk × 12 wk

**Table 3:** The main characteristics of the studies, electrical stimulation type, outcome measures, and summary.

Study	ES Modality	Outcomes and Key Finding Between-Group End/Interaction or Intervention Group (⇧ = Significant, → = no Change)	Experimental Arm	Control/Comparison	Stim. Parameters Freq (Hz) PW (µs) Current (mA)	Dose/Exposure Time (min) × Sessions (per wk × wks)	Follow-Up
tDCS							
[42]	tDCS	BBT (affected hand) ⇧ (immediate and ≥24 h; Melbourne-2 →; SCUES →	tDCS	Sham tDCS	n/a · n/a · 1.5	20 × 1	90 min
[41]	tDCS	Dynamometer, AHA → at post and 6 mo	tDCS + CIMT	Sham tDCS + CIMT	n/a · n/a · 0.7	20 × 10	6 mo
[43]	tDCS	Reduction in total movement duration ⇧; other kinematics →	tDCS + CIMT	Sham tDCS + CIMT.	n/a · n/a · 1	20 × 1	—
[56]	tDCS	3/8 AHA SDD →; 2/8 BBT SEM →; 3/8 Abilhand LMD →	tDCS+ training	—	n/a · n/a · 1.5	20 × 10	—
[46]	tDCS	Fugl-Meyer assessment ⇧, BBT ⇧, Bruininks–Oseretsky ⇧	tDCS + OT	Sham tDCS + OT	n/a · n/a · 1.5	20 × 20	—
[48]	tDCS	SHUEE (spontaneous use, grasp-release) →	tDCS + CIMT/BT	Sham tDCS + CIMT/BT	n/a · n/a · 1	20 × 13	3 mo
[47]	tDCS	AHA →, BBT →, JTHFT →	tDCS + CIMT+ Training	Sham tDCS + CIMT + Training	n/a · n/a · 1	20 × 10	6 mo
[52]	tDCS	Dynamometer tDCS-offline ⇧; sham-tDCS-offline ⇧; sham-tDCS-online ⇧; wrist ROM (ROM-W) tDCS-offline ⇧; sham-tDCS-offline ⇧; sham-tDCS-online ⇧; elbow ROM (ROM-E) tDCS-offline ⇧; sham-tDCS-offline ⇧; sham-tDCS-online ⇧	tDCS † + MVF	—	n/a · n/a · 1	20 × 1 (each, 1 wk apart) (4 sessions/conditions)	—
[50]	tDCS	BBT →	tDCS (1× mock = 3 × active + 1 × sham)	—	n/a · n/a · 1.5	20 × 3	—
FES							
[54]	FES	Microfet-2 dynamometer wrist (extensor strength: 30° ⇧, neutral ⇧, flexed 30° →; flexor strength: extended 30° ⇧, other →; JTHFT →	FES + training	—	30 · 300 · until visible contractions	5 s precontraction 3/wk × 8 wk Contractions/Session not stated	—
[51]	FES	QUEST ⇧; goniometer (wrist) ⇧ (post and 3 mo; n:7/13 missed follow-up)	FES + NDT	—	50 · 300 · 10–40	15–30. 5/wk × 3 wk	1 mo and 3 mo
[55]	FES	JTHFT (draughts ⇧, cards ⇧, objects ⇧) PC system (active wrist extension: ⇧ (not in severe contracture; n:2)), extension moment →	FES	—	30 · 300 · 10–40	30 daily × 6 wk	6 wk
TENS							
[40]	TENS	QUEST (grasp and dissociated move) ⇧	TENS + training + CIMT	Sham TENS + TOT +CIMT	100 · 200 · until sensation threshold	60, 3/wk × 8 wk	—
[39]	TENS	Dynamometer ⇧; JTHFT, ABILHAND →	TENS + training	training only	100 · 250 · 50	30, 3/wk × 8 wk	—
[44]	TENS	9HPT ⇧; QUEST (grasp) ⇧; ABILHAND →	TENS + SM-TOT + CIMT	Sham TENS + SM-TOT + CIMT	100 · 200 · until the subject felt the initial current sensation	60, 3/wk × 8 wk	—
NME							
[45]	NMES	Sphygmomanometry, UEFT ⇧ (3 and 6 mo); goniometer, 9HPT, PDMS (grasping) →	NMES + CIMT/OT	CIMT/OT	50 · 300 · ≤ 100	20 × 5/wk × 2 wk	3 and 6 mo
[53]	NMES	Daniels and Worthingham (arm flexed, all children ⇧; <4 years ⇧; ≥4 years: ⇧; arm extended, all children; <4 years ⇧; ≥4 years →) Zancolli classification (tone/hand posture) ⇧.	NMES + NDT	—	40 ·300 · 2–10	20–40 × 12 (5 wk)	3 mo
[49]	NMES	PDMS ⇧	PT + NMES	PT + KT	30 · 300 · to tolerate	20 × 3/wk × 12 wk	-

Abbreviations: AHA = assisting hand assessment; BBT = box-and-blocks test; BT = bimanual training; CIMT = constraint-induced movement therapy; FES = functional ES; JTHFT = Jebsen–Taylor hand function test; KT = kinesio taping; LMD = least measurable diff.; Melbourne: Melbourne assessment of unilateral upper limb function-2; MVF = mirror visual feedback; NDT = neurodevelopmental therapy; NMES = neuromuscular ES; OT = occupational therapy; PDMS = Peabody scales; PW = pulse width; QUEST = Quality of UE Skills Test; ROM = range of motion; SDD = substantial detectable diff.; SEM = standard error of measurement; SM-TOT = sensorimotor + task-oriented Training; SCUES: Selective Control of the Upper Extremity Scale; TOT = task-oriented training; UEFT = Upper Extremity Functional Test; 9HPT: 9-hole peg test; † tDCS vs. sham × offline vs. online mirror-visual-feedback (tDCS-offline, tDCS-online, sham tDCS-offline, sham tDCS-online).

**Table 4:** Risk-of-bias judgments for all included studies.

	[42]	[43]	[41]	[56]	[54]	[51]	[40]	[39]	[44]	[45]	[53]	[55]	[46]	[48]	[47]	[52]	[50]	[49]
Study design	Low	Low	Low	High	High	High	Low	Low	Low	Low	High	High	Low	Low	Low	Low	High	Some
Random sequence generation	Low	Low	Low	N/A	N/A	N/A	Low	Low	Low	Low	N/A	N/A	Low	Low	Low	Low	N/A	Low
Allocation concealment	Low	Low	Low	N/A	N/A	N/A	Low	Low	Low	Low	N/A	N/A	Low	Low	Low	Some	N/A	Some
Baseline differences	Some	Low	Low	Some	Some	Some	Low	Low	Low	Low	Some	Some	Low	Low	Low	Low	Some	Low
Blinding of participants	Low	Low	Low	High	High	Some	Low	Some	Low	Some	High	Some	Low	Low	Low	Low	High	Some
Blinding of personnel	Some	Low	Low	High	Some	High	Low	Some	Low	Low	High	Some	Some	Some	Low	Some	High	Some
Deviations from intended intervention	Low	Low	Low	Low	Low	Low	Low	Low	Low	Some	Low	Some	Low	Low	Low	Low	Low	Low
Effect of assignment intervention analysis	Low	Low	Low	Some	Low	Some	Low	Low	Low	Low	Some	Some	Low	Low	Low	Low	Low	Some
Concurrent intervention/unintended exposure	Low	Low	Low	Low	Low	Low	Low	Low	Low	Low	Some	Some	Low	Low	Low	Low	Low	Low
Incomplete outcome data	Low	Low	Low	Low	Low	Some	Low	Low	Low	Low	Some	Some	Low	Low	Low	Low	Some	Some
Method of measuring the outcome	Low	Low	Low	Low	Low	Low	Low	Low	Low	Low	Low	Low	Low	Low	Low	Low	Low	Low
Blinding of outcome assessor	Low	Low	Low	Some	High	Some	Some	Some	Low	Low	High	Some	Low	Low	Low	Low	Some	Low
Selective reporting	Low	Low	Low	Low	Some	Low	Low	Low	Low	Low	Some	Some	Low	Low	Low	Low	Low	Low
Overall	Some	Low	Low	High	High	High	Low	Some	Low	Some	High	High	Low	Low	Low	Some	High	Some

## Data Availability

No new data were created for this systematic review; all findings are drawn from previously published research. Further inquiries can be directed to the corresponding author(s).

## Supplementary Materials

The following supporting information can be downloaded at: https://www.mdpi.com/article/10.3390/jcm14196718/s1, Table S1: Abstract Checklist, Table S2: Checklist PRISMA 2020, Table S3: PICO Framework, Table S4: Keywords, Table S5: Modified version of the Cochrane Risk of Bias Tool 2.

## Abbreviations

The following abbreviations are used in this manuscript:

AHA	Assisting hand assessment
BBT	Box-and-blocks test
ES	Electrical stimulation
CP	Cerebral palsy
CYP	Children and young people
CIMT	Constraint-induced movement therapy
FES	Functional electrical stimulation
HCP	Hemiplegic cerebral palsy
JTHFT	Jebsen–Taylor hand function test
Melbourne-2	Melbourne assessment of unilateral upper limb function-2
NMES	Neuromuscular electrical stimulation
PDMS	Peabody developmental motor scales
QUEST	Quality of Upper Extremity Skills Test
RCTs	Randomised controlled trials
SCUES	Selective Control of the Upper Extremity Scale
TENS	Transcutaneous electrical nerve stimulation
TSCS	Transcutaneous spinal cord stimulation
tDCS	Transcranial direct current stimulation
9HPT	Nine-hole peg test
