# Supplementary material for: Effectiveness of Electrical Stimulation on Upper Limb Function in Children and Young People with Hemiplegic Cerebral Palsy: A Systematic Review
Source: J Clin Med. 2025 Sep 23;14(19):6718. doi: 10.3390/jcm14196718 (PMC12525444; doi:10.3390/jcm14196718)
Supplement: Supplementary file 1 [file jcm-14-06718-s001.zip › jcm-3757968-supplementary.pdf]

Supplementary file

Table S1: Abstract Checklist

Section and Topic	Item #	Checklist item	Reported (Yes/No)
**TITLE**			
Title	1	Identify the report as a systematic review.	Yes
**BACKGROUND**			
Objectives	2	Provide an explicit statement of the main objective(s) or question(s) the review addresses.	Yes
**METHODS**			
Eligibility criteria	3	Specify the inclusion and exclusion criteria for the review.	Yes
Information sources	4	Specify the information sources (e.g. databases, registers) used to identify studies and the date when each was last searched.	Yes
Risk of bias	5	Specify the methods used to assess risk of bias in the included studies.	Yes
Synthesis of results	6	Specify the methods used to present and synthesise results.	Yes
**RESULTS**			
Included studies	7	Give the total number of included studies and participants and summarise relevant characteristics of studies.	Yes
Synthesis of results	8	Present results for main outcomes, preferably indicating the number of included studies and participants for each. If meta-analysis was done, report the summary estimate and confidence/credible interval. If comparing groups, indicate the direction of the effect (i.e. which group is favoured).	Yes
**DISCUSSION**			
Limitations of evidence	9	Provide a brief summary of the limitations of the evidence included in the review (e.g. study risk of bias, inconsistency and imprecision).	Yes
Interpretation	10	Provide a general interpretation of the results and important implications.	Yes
**OTHER**			
Funding	11	Specify the primary source of funding for the review.	Yes
Registration	12	Provide the register name and registration number.	Yes

Table S2: Checklist PRISMA 2020

Section and Topic	Item #	Checklist item	Location where item is reported
**TITLE**			
Title	1	Identify the report as a systematic review.	1
**ABSTRACT**			
Abstract	2	See the PRISMA 2020 for Abstracts checklist.	1
**INTRODUCTION**			
Rationale	3	Describe the rationale for the review in the context of existing knowledge.	2-3
Objectives	4	Provide an explicit statement of the objective(s) or question(s) the review addresses.	3
**METHODS**			
Eligibility criteria	5	Specify the inclusion and exclusion criteria for the review and how studies were grouped for the syntheses.	3-4
Information sources	6	Specify all databases, registers, websites, organisations, reference lists and other sources searched or consulted to identify studies. Specify the date when each source was last searched or consulted.	5-6
Search strategy	7	Present the full search strategies for all databases, registers and websites, including any filters and limits used.	3-4 - Table S4 -
Selection process	8	Specify the methods used to decide whether a study met the inclusion criteria of the review, including how many reviewers screened each record and each report retrieved, whether they worked independently, and if applicable, details of automation tools used in the process.	3-4
Data collection process	9	Specify the methods used to collect data from reports, including how many reviewers collected data from each report, whether they worked independently, any processes for obtaining or confirming data from study investigators, and if applicable, details of automation tools used in the process.	4
Data items	10a	List and define all outcomes for which data were sought. Specify whether all results that were compatible with each outcome domain in each study were sought (e.g. for all measures, time points, analyses), and if not, the methods used to decide which results to collect.	3-4
	10b	List and define all other variables for which data were sought (e.g. participant and intervention characteristics, funding sources). Describe any assumptions made about any missing or unclear information.	4
Study risk of bias assessment	11	Specify the methods used to assess risk of bias in the included studies, including details of the tool(s) used, how many reviewers assessed each study and whether they worked independently, and if applicable, details of automation tools used in the process.	4-5
Effect measures	12	Specify for each outcome the effect measure(s) (e.g. risk ratio, mean difference) used in the synthesis or presentation of results.	-
Synthesis methods	13a	Describe the processes used to decide which studies were eligible for each synthesis (e.g. tabulating the study intervention characteristics and comparing against the planned groups for each synthesis (item #5)).	3-4
	13b	Describe any methods required to prepare the data for presentation or synthesis, such as handling of missing summary statistics, or data conversions.	-
	13c	Describe any methods used to tabulate or visually display results of individual studies and syntheses.	3-4
	13d	Describe any methods used to synthesize results and provide a rationale for the choice(s). If meta-analysis was performed, describe the model(s), method(s) to identify the presence and extent of statistical heterogeneity, and software package(s) used.	
	13e	Describe any methods used to explore possible causes of heterogeneity among study results (e.g. subgroup analysis, meta-regression).	4
	13f	Describe any sensitivity analyses conducted to assess robustness of the synthesized results.	-
Reporting bias assessment	14	Describe any methods used to assess risk of bias due to missing results in a synthesis (arising from reporting biases).	3-4
Certainty assessment	15	Describe any methods used to assess certainty (or confidence) in the body of evidence for an outcome.	-
**RESULTS**			
Study selection	16a	Describe the results of the search and selection process, from the number of records identified in the search to the number of studies included in the review, ideally using a flow diagram.	5-6

Section and Topic	Item #	Checklist item	Location where item is reported
	16b	Cite studies that might appear to meet the inclusion criteria, but which were excluded, and explain why they were excluded.	8
Study characteristics	17	Cite each included study and present its characteristics.	8-14
Risk of bias in studies	18	Present assessments of risk of bias for each included study.	13-14
Results of individual studies	19	For all outcomes, present, for each study: (a) summary statistics for each group (where appropriate) and (b) an effect estimate and its precision (e.g. confidence/credible interval), ideally using structured tables or plots.	10-12
Results of syntheses	20a	For each synthesis, briefly summarise the characteristics and risk of bias among contributing studies.	12-14
	20b	Present results of all statistical syntheses conducted. If meta-analysis was done, present for each the summary estimate and its precision (e.g. confidence/credible interval) and measures of statistical heterogeneity. If comparing groups, describe the direction of the effect.	-
	20c	Present results of all investigations of possible causes of heterogeneity among study results.	8-14
	20d	Present results of all sensitivity analyses conducted to assess the robustness of the synthesized results.	-
Reporting biases	21	Present assessments of risk of bias due to missing results (arising from reporting biases) for each synthesis assessed.	-
Certainty of evidence	22	Present assessments of certainty (or confidence) in the body of evidence for each outcome assessed.	-
**DISCUSSION**			
Discussion	23a	Provide a general interpretation of the results in the context of other evidence.	
	23b	Discuss any limitations of the evidence included in the review.	
	23c	Discuss any limitations of the review processes used.	
	23d	Discuss implications of the results for practice, policy, and future research.	
**OTHER INFORMATION**			
Registration and protocol	24a	Provide registration information for the review, including register name and registration number, or state that the review was not registered.	14-17
	24b	Indicate where the review protocol can be accessed, or state that a protocol was not prepared.	-
	24c	Describe and explain any amendments to information provided at registration or in the protocol.	-
Support	25	Describe sources of financial or non-financial support for the review, and the role of the funders or sponsors in the review.	18
Competing interests	26	Declare any competing interests of review authors.	
Availability of data, code and other materials	27	Report which of the following are publicly available and where they can be found: template data collection forms; data extracted from included studies; data used for all analyses; analytic code; any other materials used in the review.	

Table S3: PICO Framework

PICOS	
**Population**	Children and Young people (2 - 21 years old), female and/or male, Hemiplegic Cerebral Palsy
**Intervention**	**Electrical stimulation:**<br>1. Transcutaneous electrical nerve stimulation (TENS)<br>2. Neuromuscular electrical stimulation (NMES)<br>3. Functional electrical stimulation (FES)<br>4. Transcranial direct current stimulation (tDCS)<br>5. Transcutaneous spinal cord stimulation (TSCS).
**Comparison**	Studies utilising ES pules physical therapy, occupational therapy, or ES alone.<br><br>Physical and occupational therapy: including exercises (strength, range of motion, stretching), functional exercises (sensorimotor training, task-oriented training), facilitation and inhibition of muscle tone (neurodevelopmental treatment).
**Outcome**	Upper limb and hand function outcomes (including fine and gross motor skills, range of motion, muscle strength, functional grip and release, isolated finger movements, protective responses, weight-bearing capacity, object manipulation, movement fluidity, placement accuracy, and performance in daily activities)
**Study**	English studies published of any design were included (except case studies with fewer than 3 participants)

Table S4: Keywords

PICO	
**Population**	Cerebral Palsy OR CP OR Children with Cerebral Palsy OR Spastic Cerebral Palsy OR Spastic Children OR Children with Spastic Cerebral Palsy OR Spastic Hemiplegia OR Hemiplegic OR Hemiplegic Patients OR Patient with Hemiplegia OR Children with hemiplegia OR Children with Unilateral Cerebral Palsy OR Unilateral Cerebral Palsy OR Children with Unilateral Spastic Cerebral Palsy OR Young with Physical Disabilities OR Paediatric Cases OR Motor Delay OR Acquired Brain Injury
**Intervention**	Electrical Stimulation OR ES OR Transcutaneous Electrical Stimulation OR TCES OR Transcutaneous Electrical Nerve Stimulation OR TENS OR Functional Electrical Stimulation OR FES OR Transcranial Direct Current Stimulation OR tDCS OR Neuromuscular Electrical Stimulation OR NMES OR Transcutaneous Spinal Cord Electrical Stimulations OR tSCS OR Spinal Cord Stimulations OR SCS OR Spinal Cord Electrical Stimulations OR SCES OR Transcutaneous Spinal Stimulation OR TSS OR Painless Transcutaneous Electrical Enabling Motor Control OR pcEmc OR Transcutaneous Electrical Stimulation of the Spinal Cord OR TESS OR Transcutaneous Enabling Motor Control OR tEmc OR Transcutaneous Spinal Direct Current Stimulation OR tsDCS OR Somatosensory Electrical Stimulation OR SES OR Peripheral Nerve Stimulation or PNS
**Comparison Outcome**	Hand Function OR Arm Function OR Upper Limb Function OR Upper Extremity Function OR Head Control OR Hand OR Arm OR Upper Limb OR Fine Motor Skills OR Motor Skills OR Grasping OR Functional Skills OR Skill Acquisition OR Motor Learning OR Functional Independency OR Rehabilitation OR Hand Rehabilitation OR Physiotherapy OR Physical Rehabilitation
**Database**	CINAHL, Ovid MEDLINE, Cochrane, EMBASE, Web of Science, PsycINFO & PUBMED, Pedro and Scopus
**Note**	Keywords were applied across all databases. Where available, filters were used to limit results to English-language, human studies, and clinical or experimental studies; review articles were excluded. Boolean operators were used according to each platform's advanced search guidance. For PEDro, the search strategy was implemented as follows:<br>1. Abstract & title: cerebral palsy; therapy: electrotherapies, heat, cold; body part; body part: head and neck<br>2. Abstract & title: cerebral palsy; therapy: electrotherapies, heat, cold; body part: upper arm, shoulder or shoulder girdle<br>3. Abstract & title: cerebral palsy; therapy: electrotherapies, heat, cold; body part: forearm or elbow<br>4. Abstract & title: cerebral palsy; therapy: electrotherapies, heat, cold; body part: hand or wrist

Table S5: *Modified version of the Cochrane Risk of Bias Tool 2.*

Bias domain	Source of bias	Support for judgement	Review author's judgement (assessed as high, low or some concern of bias, or non-applicable)
**Domain 1. Risk of bias arising from the randomisation process**	Type of study design	Describe whether the type of study design or analysis control accounts for important confounding and modifying variables through matching, stratification, multivariable analysis or other approaches.	Selection bias (biased allocation to intervention) due to the type of study design employed.
	Random sequence generation[1]	Describe the method used to generate the allocation sequence in sufficient detail to allow an assessment of whether it should produce comparable groups.	Selection bias (biased allocation to interventions) due to inadequate generation of a randomised sequence.
	Allocation concealment[1]	Describe the method used to conceal the allocation sequence in sufficient detail to determine whether intervention allocations could have been foreseen before or during enrolment.	Selection bias (biased allocation to interventions) due to inadequate concealment of allocations before assignment.
	Baseline differences	Describe whether there were any baseline differences between intervention groups that would suggest there was a problem with the randomisation process.	Selection bias (biased allocation to interventions) due to baseline differences between intervention groups.
**Domain 2. Risk of bias due to deviations from the intended intervention**	Blinding of participants	Describe all measures used, if any, to blind trial participants from knowledge of the intervention they received. Provide any information relating to whether the intended blinding was effective.	Performance bias due to knowledge of the allocated interventions by participants during the study.
	Blinding of people delivering the intervention	Describe all measures used, if any, to blind trial researchers from knowledge of the intervention a participant received. Provide any information relating to whether the intended blinding was effective.	Performance bias due to knowledge of the allocated interventions by the people delivering the intervention.
	Deviations from intended intervention	Describe if there were changes from assigned intervention due to the trial context. Provide any information relating to whether the deviations are likely to have affected the outcome and whether the deviations were balanced between intervention groups.	Performance bias due to deviations from the intended intervention.
	Effect of assignment intervention analysis	Describe if any appropriate analyses were used to estimate the effect of assignment to intervention. Provide information relating to whether there was potential for substantial impact (on the result) of the failure to analyse participants in the group to which they were randomised.	Performance bias due to inadequate analysis to estimate the effect of assignment to intervention.
	Concurrent intervention/ unintended exposure	Describe whether any concurrent interventions or unintended exposures occurred which could have biased the results.	Performance bias due to exposure to factors other than the allocated intervention.
**Domain 3. Risk of bias due to missing outcome data**	Incomplete outcome data[2]	Describe the completeness of outcome data. Provide any information relating to evidence that the result was not biased by missing outcome data, and how likely it is that missingness in the outcome depends on its true value.	Attrition bias due to amount, nature, or handling of incomplete outcome data
**Domain 4. Risk of bias in measurement of the outcome**	Method of measuring the outcome	Describe how appropriate the method of measuring the outcome was. Provide any information relating to whether the measurement of the outcome could have differed between groups.	Detection bias due to method of measuring the outcome.
	Blinding of outcome assessment[2]	Describe all measures used, if any, to blind outcome assessment from knowledge of the intervention a participant received. Provide any information relating to whether the intended blinding was effective.	Detection bias due to knowledge of the allocated interventions by outcome assessment.
**Domain 5. Risk of bias in selection of the reported result**	Selective reporting	If a protocol is available state whether all outcomes specified in the protocol were included in the published report. If no protocol is available state whether all outcomes specified in the methods were included in the results and whether all key outcomes for the study area were included.	Reporting bias due to selective outcome reporting.
